# Immunomodulation via MyD88-NFκB Signaling Pathway from Human Umbilical Cord-Derived Mesenchymal Stem Cells in Acute Lung Injury

**DOI:** 10.3390/ijms23105295

**Published:** 2022-05-10

**Authors:** Kang-Hsi Wu, Ju-Pi Li, Wan-Ru Chao, Yi-Ju Lee, Shun-Fa Yang, Ching-Chang Cheng, Yu-Hua Chao

**Affiliations:** 1 Department of Pediatrics, Chung Shan Medical University Hospital, Taichung 402, Taiwan; cshy1903@gmail.com (K.-H.W.); d888203@gmail.com (J.-P.L.); 2 Department of Pediatrics, School of Medicine, Chung Shan Medical University, Taichung 402, Taiwan; 3 Department of Pathology, School of Medicine, Chung Shan Medical University, Taichung 402, Taiwan; littleuni@hotmail.com (W.-R.C.); jasmine.lyl@gmail.com (Y.-J.L.); 4 Department of Pathology, Chung Shan Medical University Hospital, Taichung 402, Taiwan; 5 Institute of Medicine, Chung Shan Medical University, Taichung 402, Taiwan; ysf@csmu.edu.tw; 6 Department of Medical Research, Chung Shan Medical University Hospital, Taichung 402, Taiwan; 7 Laboratory Animal Service Center, Office of Research and Development, China Medical University, Taichung 402, Taiwan; showshow330907@gmail.com; 8 Department of Clinical Pathology, Chung Shan Medical University Hospital, Taichung 402, Taiwan

**Keywords:** acute lung injury, immunomodulation, mesenchymal stem cells, myeloid differentiation factor 88, Toll-like receptors

## Abstract

Excess inflammatory processes play a key detrimental role in the pathophysiology of acute lung injury (ALI). Mesenchymal stem cells (MSCs) were reported to be beneficial to ALI, but the underlying mechanisms have not been completely understood. The present study aimed to examine the involvement of MyD88–NFκB signaling in the immunomodulation of MSCs in mice with lipopolysaccharides (LPS)-induced ALI. We found that serum concentrations of IL-6, TNF-α, MCP-1, IL-1β, and IL-8 were significantly decreased at 6 h after LPS-induced ALI in the MSC group (*p* < 0.05). For each of the five cytokines, the serum concentration of each individual mouse in either group declined to a similar level at 48 h. The intensity of lung injury lessened in the MSC group, as shown by histopathology and lung injury scores (*p* < 0.001). The expressions of MyD88 and phospho-NFκB in the lung tissue were significantly decreased in mice receiving MSCs as measured by Western blotting and immunohistochemistry. Our data demonstrated that human umbilical cord-derived MSCs could effectively alleviate the cytokine storm in mice after LPS-induced ALI and attenuated lung injury. Firstly, we documented the correlation between the down-regulation of MyD88–NFκB signaling and immunomodulatory effects of MSCs in the situation of ALI.

## 1. Introduction

Diseases of the respiratory tract are among the leading causes of death in the world population. Acute lung injury (ALI) is characterized by rapid onset of inflammatory infiltrates in the lungs in response to various insults and can progress into acute respiratory distress syndrome, respiratory failure, and even death. Excess inflammatory reactions play a key detrimental role in the pathophysiology of ALI [1]. Despite significant advances in the management of the disease, morbidity and mortality remain high [2,3]. Novel and effective therapeutic strategies focusing on the interruption of early events to prevent disease progress are warranted.

Mesenchymal stem cells (MSCs) are considered a promising platform for cell-based therapy. Given their immunomodulatory properties, MSCs are attractive candidates to manage a variety of clinical diseases with aberrant immune responses [4,5]. Previously published data have shown the benefits of MSC administration in animal models of ALI, and the anti-inflammatory properties of MSCs may contribute to their protective role [6]. However, it remains unclear by which mechanisms these cells influence immune cells and ameliorate lung injury.

Toll-like receptors (TLRs) are crucial to immediate host responses in the early phase of infection and the linkage of innate and adaptive immunity [7,8,9]. There are eleven members of the TLR family identified in mammals. Ligand recognition by TLRs results in the recruitment of myeloid differentiation factor 88 (MyD88) and subsequently the activation of nuclear factor-κB (NFκB) [10,11]. Through MyD88–NFκB signaling, sensing the presence of infections can trigger the production and secretion of various inflammatory cytokines. The association between deficient NFκB activation and subsequent immunosuppression was found [12,13], suggesting that inhibition of massive NFκB could reduce inflammatory reactions. To date, there are no reports regarding the effects of MSCs on this signaling in the context of ALI. In the present study, we aimed to investigate the immunomodulatory effects of MSCs on mice with lipopolysaccharides (LPS)-induced ALI as well as to evaluate the role of MyD88-NFκB signaling in this circumstance.

## 2. Results

### 2.1. Characterization of MSCs

**Figure 1:**
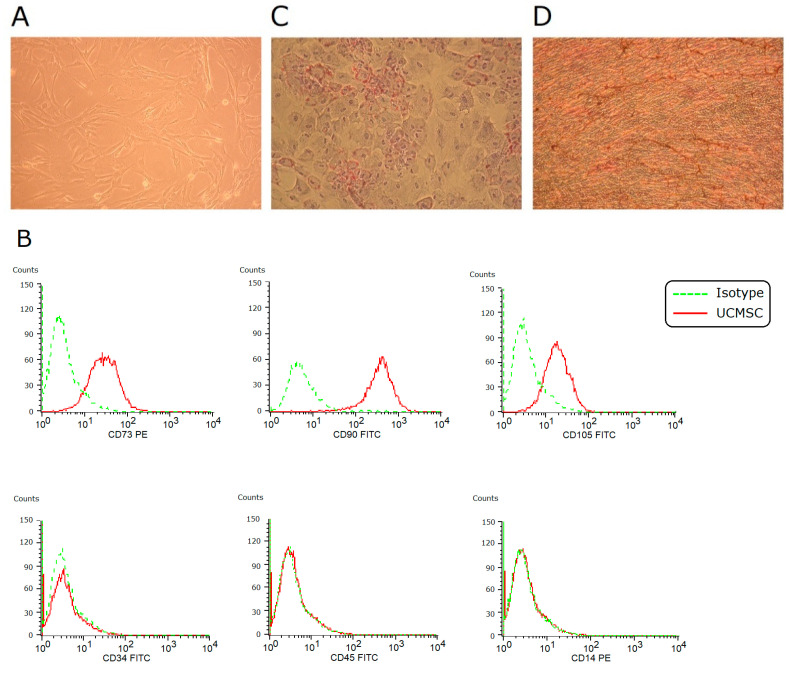
Characterization of UCMSCs. (**A**) In vitro culture, MSCs showed a spindle-shaped fibroblastic morphology (×100). (**B**) Using flow cytometry, these cells were positive for CD73, CD90, and CD105, and negative for CD34, CD45, and CD14. (**C**) Under 2-week adipogenic induction, differentiation into adipocytes was achieved (Oil red O staining, ×100). (**D**) Under 2-week osteogenic induction, differentiation into osteocytes was achieved (von Kossa staining, ×100).

In vitro culture, umbilical cord-derived MSCs (UCMSCs) adhered to plates and showed a spindle-shaped morphology (Figure 1A). They expressed CD73, CD90, and CD105, and they were negative for CD34, CD45, and CD14 (Figure 1B). Under induction conditions, they achieved adipogenesis and osteogenesis (Figure 1C,D). These findings fulfilled the criteria of the International Society for Cellular Therapy to define human MSCs.

### 2.2. MSCs Alleviating Cytokine Storm after LPS-Induced ALI

**Figure 2:**
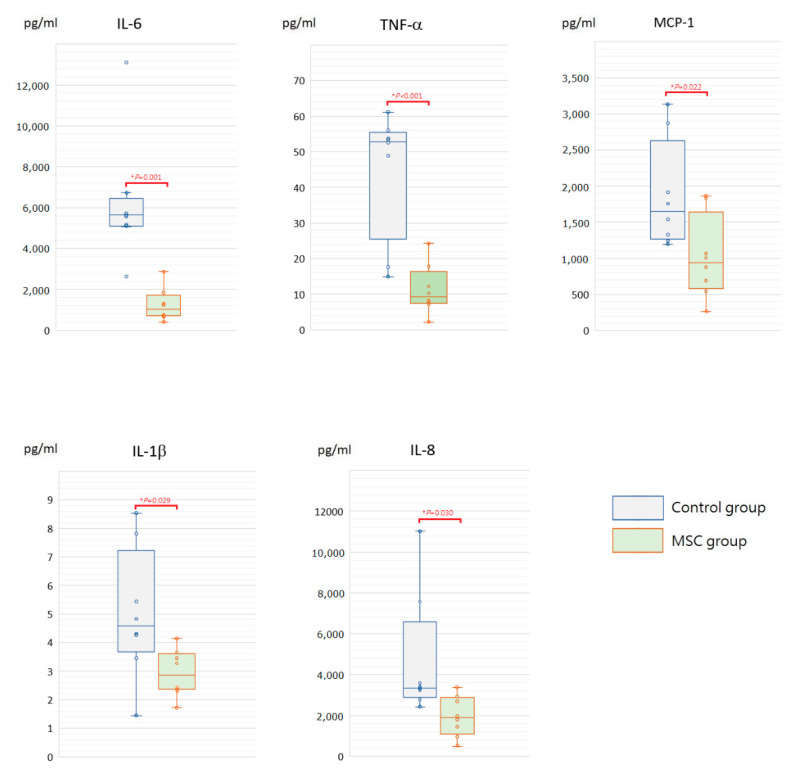
Boxplots of serum inflammatory cytokine levels at 6 h after LPS-induced ALI. Compared to the control group, serum levels of IL-6, TNF-α, MCP-1, IL-1β, and IL-8 were significantly decreased in the MSC group. The box is drawn from the first quartile to the third quartile with a horizontal line in the middle to denote the median. The whiskers extending from the box indicate variability outside the upper and lower quartiles. Each circle represents the concentration of each animal. *n* = 8 mice/group.

**Figure 3:**
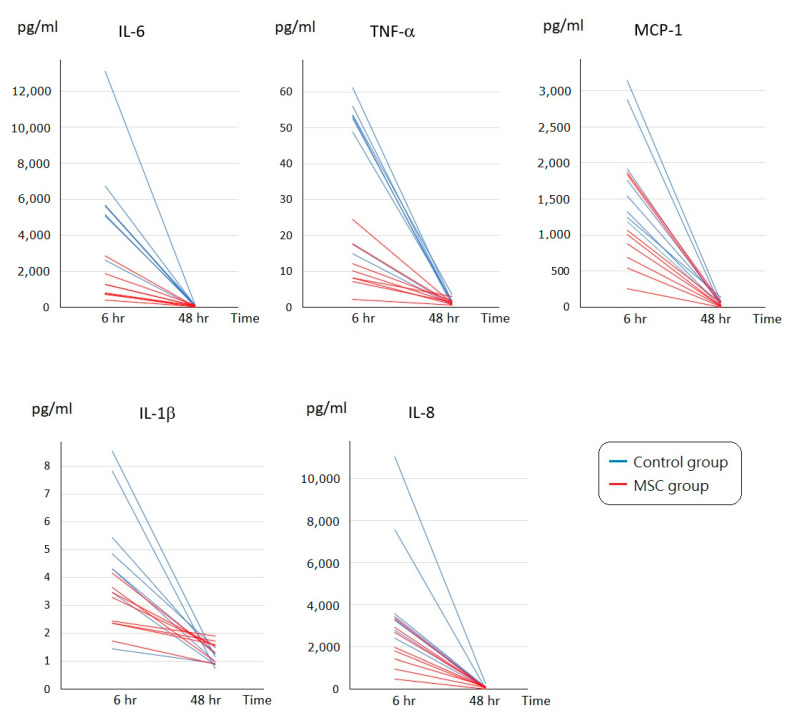
Time-series changes on circulating inflammatory cytokine levels of each individual mouse at 6 and 48 h after LPS-induced ALI. For IL-6, TNF-α, MCP-1, IL-1β, or IL-8, the serum concentration of each individual mouse in either group declined to a similar level at 48 h after LPS challenge. *n* = 8 mice/group.

To assess the initial changes of circulating inflammatory cytokine profiles after LPS-induced ALI, serum concentrations of interleukin (IL)-6, tumor necrosis factor-α (TNF-α), monocyte chemoattractant protein-1 (MCP-1), IL-1β, and IL-8 were measured at 6 h after LPS challenge (Figure 2). Compared to mice of the control group receiving phosphate-buffered saline (PBS) only after LPS-induced ALI, all of these cytokine levels were significantly lower in mice of the MSC group (*p* < 0.05). In addition, we measured IL-6, TNF-α, MCP-1, IL-1β, and IL-8 in blood serum obtained after sacrifice. For each of the five cytokines, the serum concentration of each individual mouse in either group declined to a similar level at 48 h after LPS challenge (Figure 3). We speculated that MSC administration could alleviate the cytokine storm that occurred around 6 h after LPS-induced ALI. It gradually cooled in 48 h despite MSC administration or not.

### 2.3. MSCs Attenuating Lung Injury after LPS-Induced ALI

**Figure 4:**
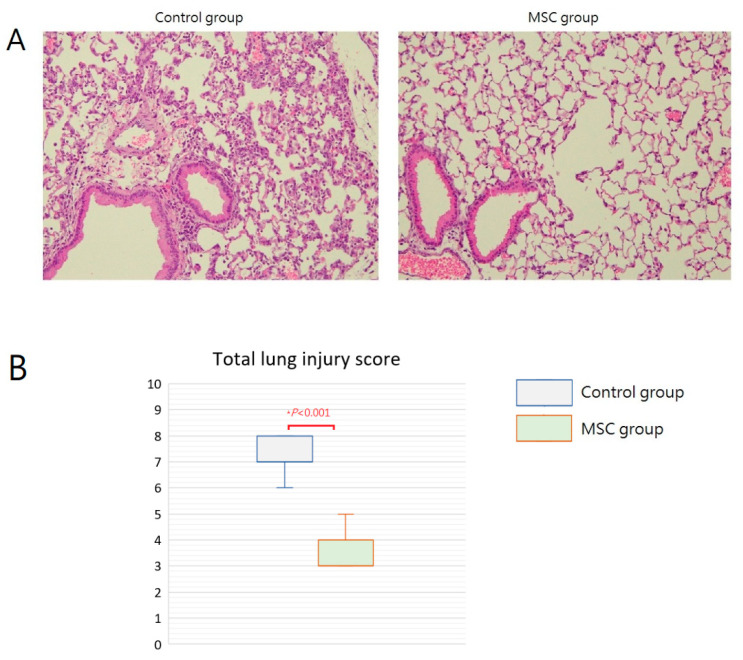
Effects of MSC administration on morphology of the lungs at 48 h after LPS-induced ALI. (**A**) Microscopic histopathology of the lung tissue (hematoxylin and eosin, ×200). (**B**) Boxplots of total lung injury scores. There was a significant difference between the two groups.

Grossly, multiple focal hemorrhagic spots with edematous change can be found in the lungs of mice in the control group. In contrast, the lungs of mice in the MSC group appeared grossly normal. Microscopically, the lung tissue was significantly injured with the presence of septal edema and massive inflammatory cell infiltration in the control group compared with that in the MSC group (Figure 4A). The mean lung injury score was 7.25 and 3.50 in mice of the control group and the MSC group, respectively (Figure 4B). Most evidently, MSC-treated mice demonstrated less intra-alveolar macrophage and neutrophil infiltration (Table 1). These findings provided beneficial evidence for lung protection from MSC administration in mice with LPS-induced ALI.

### 2.4. MSCs Restraining Elevation of Inflammatory Cytokine Levels in the BALF after LPS-Induced ALI

**Figure 5:**
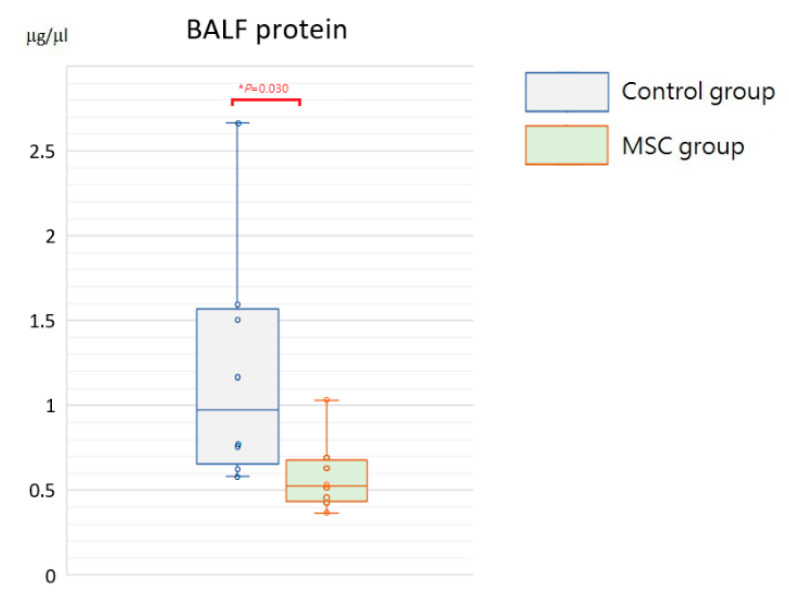
Boxplots of protein levels in the BALF at 48 h after LPS-induced ALI. Protein levels in the BALF were significantly higher in the control group. Each circle represents the concentration of each animal. *n* = 8 mice/group.

**Figure 6:**
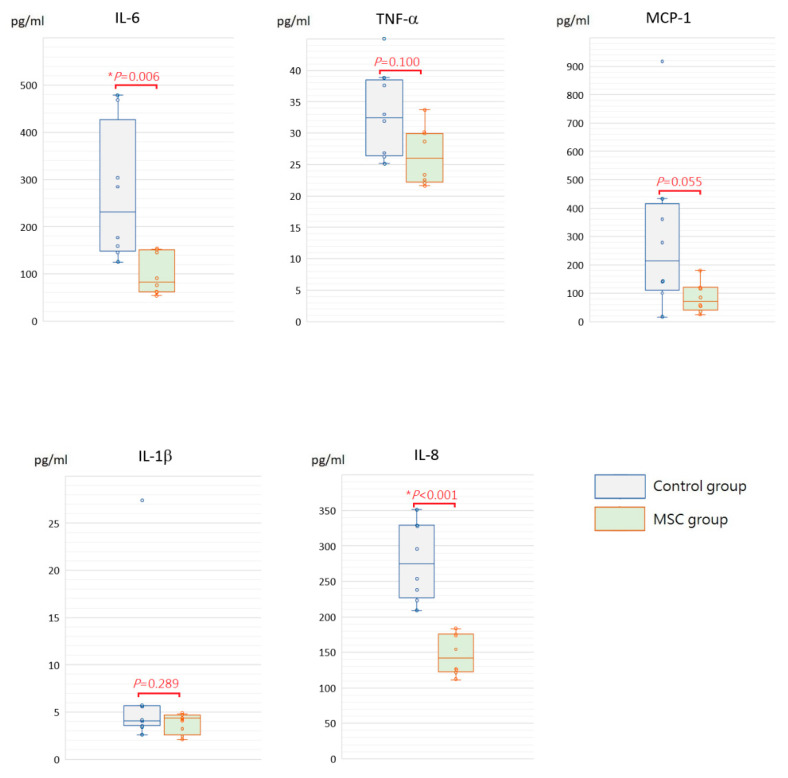
Boxplots of inflammatory cytokine levels in the BALF at 48 h after LPS-induced ALI. Compared to the control group, concentrations of IL-6 and IL-8 in the BALF were significantly decreased in the MSC group. Although not reaching statistical significance, levels of TNF-α, MCP-1, and IL-1β trended lower in the MSC group. Each circle represents the concentration of each animal. *n* = 8 mice/group.

Being a marker of endothelial and epithelial permeability, protein levels in the bronchoalveolar lavage fluid (BALF) are linked to the status of inflammation in lung tissue and thought to be able to exacerbate lung injury. As shown in Figure 5, concentrations of BALF protein were significantly reduced in mice receiving MSCs after LPS challenge compared to those receiving PBS only (*p* = 0.030). Furthermore, we measured inflammatory cytokine levels, including IL-6, TNF-α, MCP-1, IL-1β, and IL-8, in the BALF. Compared to mice of the control group, concentrations of IL-6 and IL-8 were significantly decreased in mice receiving MSCs after LPS challenge (Figure 6). Although not reaching statistical significance, levels of TNF-α, MCP-1, and IL-1β trended lower in mice of the MSC group. Our results suggested that MSC administration could restrain the elevation of these cytokine concentrations in the BALF and thus ameliorate acute inflammation in mice with LPS-induced ALI.

### 2.5. MyD88-NFκB Signaling Associated with Immunomodulation from MSCs

**Figure 7:**
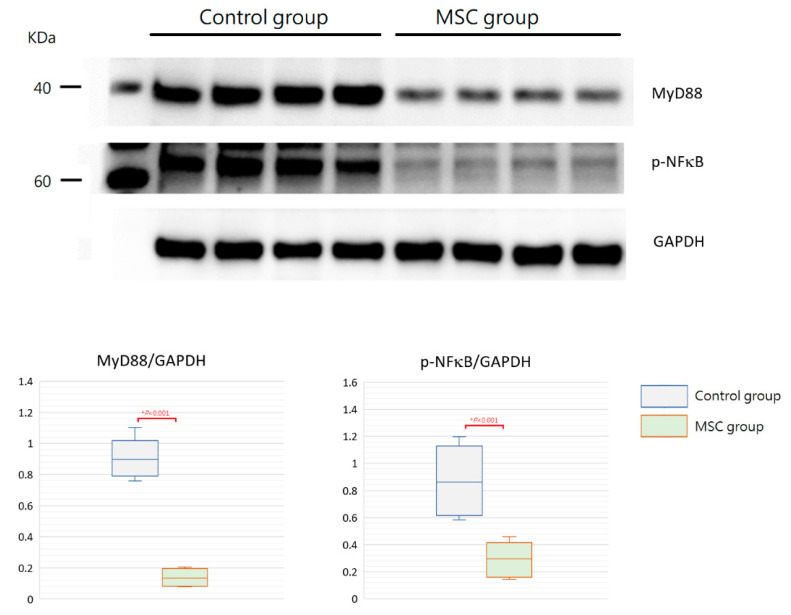
Western blot analysis for MyD88 and phospho-NFκB in the lung tissue at 48 h after LPS-induced ALI. As shown with boxplots, expressions of MyD88 and phospho-NFκB were decreased significantly in the MSC group. GAPDH was used as the loading control. *n* = 4 mice/group.

**Figure 8:**
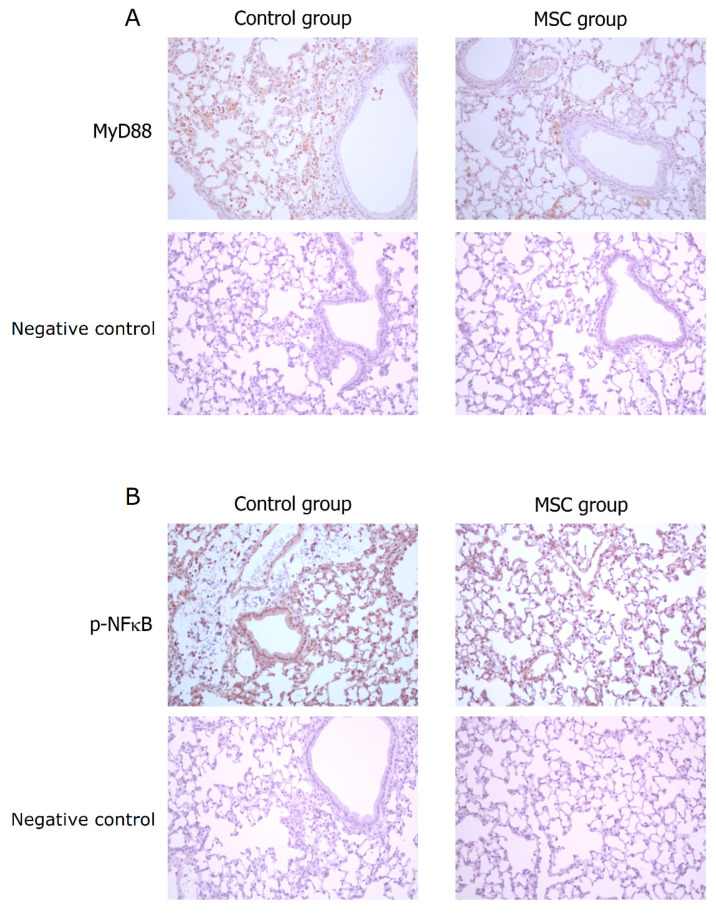
Immunohistochemical analysis for MyD88 and phospho-NFκB in the lung tissue at 48 h after LPS-induced ALI (×200). (**A**) Compared to the control group, the percentage of MyD88-positive inflammatory cells in the lung tissue as well as the intensity of staining for MyD88 within the inflammatory cells was decreased in the MSC group. (**B**) Consistently, the expression of phospho-NFκB was lower in the MSC group.

TLR-mediated reactions are important for innate immunity. To evaluate the involvement of their activation in mice with LPS-induced ALI receiving MSCs, we detected expressions of MyD88 and phospho-NFκB in the lung tissue obtained after sacrifice by Western blot analysis and immunohistochemistry. As illustrated in Figure 7, the expression of MyD88 protein was significantly decreased in mice receiving MSCs compared to those receiving PBS only (*p* < 0.001). Consistently, the downstream-activated transcription factor, phospho-NFκB, expressed lower as measured by Western blotting in mice of the MSC group (*p* < 0.001). As shown by immunohistochemical analysis in Figure 8, the percentage of MyD88-positive inflammatory cells in the lung tissue as well as the intensity of staining for MyD88 within the inflammatory cells was decreased in mice receiving MSCs after LPS challenge compared to mice receiving PBS only. As expected, the expression of phospho-NFκB was consistently lower in mice of the MSC group. These results implicated that MSCs may exert their immunomodulatory influence on mice with LPS-induced ALI via downregulation of the MyD88–NFκB signaling pathway.

## 3. Discussion

ALI is characterized by inflammation, cytokine production, neutrophil accumulation, and rapid alveolar damage. Given that excessive inflammatory response plays a key detrimental role in the development of ALI [1], the important work needs to focus on the interruption of early events in disease pathogenesis. With profound immunomodulatory properties, MSCs have emerged as a promising therapeutic strategy for various inflammatory diseases [4,5]. In the present study, a well-characterized animal model of LPS-induce ALI was used to mimic human ALI and to stimulate host inflammatory responses. We found that the intraperitoneal administration of human UCMSCs effectively ameliorated the surge of inflammatory cytokines after LPS challenge and attenuated lung injury in mice with ALI. For the first time, we demonstrated that down-regulation of the MyD88–NFκB signaling pathway contributed to the immunomodulatory effects of MSCs in the situation of ALI.

Adequate inflammatory reactions are essential for protecting the body from invasion of infectious pathogens. However, an uncontrollable pulmonary inflammation caused by large amounts of inflammatory cells and cytokines leads to the development of ALI, and the degree of acute inflammation is highly associated with the outcome of human ALI [14]. In the present study, serum levels of inflammatory cytokines, including IL-6, TNF-α, MCP-1, IL-1β, and IL-8, were measured at 6 h and 48 h after LPS-induced ALI. All these cytokine levels were significantly lower at 6 h in mice receiving MSCs, implicating that MSCs effectively ameliorated the cytokine storm occurred around 6 h after LPS challenge. Although the cytokine concentration in each individual mouse of either group declined to a similar level at 48 h for each of the five cytokines, as shown in Figure 3, the occurrence of cytokine storm did cause harm in the lungs already. The intensity of pathological changes observed grossly and microscopically was increased in mice of the control group, and there were higher lung injury scores as well. Notably, the infiltration of macrophages and neutrophils in the intra-alveolar, peribronchial, and perivascular space was significantly increased. It is known that inflammatory cell migration and infiltration into the site of inflammation is extremely important in the tissue damage of lung injury [15,16]. Consistently, protein levels in the BALF along with the inflammatory cytokine concentrations were higher in mice receiving PBS only after LPS-induced ALI, suggesting the increase in the leakage of fluid and macromolecules due to alveolar capillary endothelial injury. As known, vascular permeability is the most important initial cause of ALI and related to the outcome [17]. Taken together, our data suggested that MSCs blocked the recruitment of excess inflammatory cells into the lungs and attenuated the surge of inflammatory cytokines in mice with ALI. With the effective modulation of early inflammatory conditions, MSCs lessened the deterioration of lung injury.

MSCs possess profound immunomodulatory effects [18,19,20]. Various studies have showed beneficial effects of MSCs in animal models of ALI, and the anti-inflammatory properties of MSCs may contribute to their protective role [6]. Our present study and previous investigations demonstrated that the vast majority of infiltrating cells found in ALI were neutrophils, and the degree of infiltration was significantly decreased in MSC-treated animals [21,22,23,24]. The myeloperoxidase activity, which is an enzyme marker of neutrophil accumulation and activity, was found to be decreased in animals with ALI receiving MSC transplantation [21,22,23,24]. Danchuk et al. reported that the up-regulated expression of TNF-α-induced protein 6 was highly induced in MSCs in response to lung injury and resulted in a decrease in neutrophil accumulation and lung damage [23]. These data implicated that MSCs protected the lungs from acute inflammation primarily by preventing the recruitment and activation of neutrophils. However, the mechanisms involved are still being elucidated.

TLRs are the first-line effector molecule in response to infections, and they also act as an important bridge between innate and adaptive immune response [7,8,9]. Ligand recognition by TLRs triggers the recruitment of MyD88 and subsequently the nuclear translocation of the transcription factor NFκB [10,11]. The activation of MyD88–NFκB signaling leads to the production of a variety of inflammation-associated cytokines, and therefore, it is thought to be important in regulating inflammatory reactions. Because MSCs are potent immunomodulators to affect nearly all cell types of the immune system [18,19,20], the MyD88–NFκB signaling pathway may participate in MSC-mediated immunomodulation to a certain degree. Our previous studies reported the involvement of MyD88–NFκB signaling in immunomodulatory effects from MSCs in animal models of sepsis and systemic lupus erythematosus [25,26]. We demonstrated great benefits of MSC administration to survival in septic mice and disease control in mice with systemic lupus erythematosus. The beneficial effects from MSCs may be because the MSC administration brought the chaotic and hyper-inflammatory immune responses back into balance and consequently ameliorated self-tissue damage.

TLRs are common immune molecules to recognize bacterial pathogens during lower respiratory tract infections and play a crucial role in neutrophil sequestration in the lungs [27]. In addition to a variety of invading microbes, TLRs are capable of sensing endogenous molecules released after cell damage as well. Being a member of pattern recognition receptors in both infectious and noninfectious lung diseases, the engagement of TLRs is the prerequisite for the initiation of immune responses, which can be beneficial or detrimental to the host [28]. In the present study, we found that expression levels of MyD88 and phospho-NFκB in the lung tissue were significantly decreased in mice receiving MSCs after LPS-induced ALI. Along with the altered cytokine profiles in the serum and BALF, we speculated that down-regulated MyD88–NFκB signaling may contribute to the immunomodulatory reactions and lung protection from MSCs in mice with ALI. To the best of our knowledge, this is the first report regarding the involvement of MyD88–NFκB signaling in the immunomodulation of MSCs in an animal model of ALI. As pattern recognition receptors and their signaling pathways represent promising targets for therapeutic interventions in various lung diseases [28], MSC transplantation can be a potential treatment for ALI in humans.

MSCs have been demonstrated as an effective strategy to modulate inflammation while enhancing bacterial clearance, reducing organ damage, and improving survival in various animal models of ALI. Efficacy was maintained across different types of MSC sources and delivery approaches [6]. A variety of insults, infectious and noninfectious, can trigger pulmonary inflammation and result in the development of ALI. Among them, infection is the most common cause and leads to worse outcomes compared to noninfectious etiologies [29]. In clinical practice, it is extremely important to prevent the spread of pathogens in the management of patients with infectious diseases. Compared with other delivery routes such as intratracheal, the systemic administration of MSCs via an intravenous route is much safer for the environment and care providers. For clinical use, another important issue to note is the origin of MSCs. A broad spectrum of tissues has been identified as resources for MSCs, but autologous MSCs may not be a suitable source for cell therapy in many clinical situations. Umbilical cords are rich in MSCs, which can be easily collected and cultured without ethical problems [30]. Additionally, UCMSCs were found to have higher proliferative potential in vitro [31], indicating their advantages of rapid expansion and consequent clinical application. In humans, we found that UCMSCs could promote hematopoietic engraftment after hematopoietic stem cell transplantation and treat refractory graft-versus-host disease effectively and safely [32,33,34]. To act like in clinical use, we examined the effects of intraperitoneal delivery of UCMSCs on mice with LPS-induced ALI in the present study.

There are several limitations in the present study. First, there was no control group of untreated animals. Although important, the number of control groups and the intervention of animals in the control groups were quite different in animal models of ALI. The more control groups, the more solid information can be drawn in the study. However, there are a variety of considerations. In the literature, data of untreated group were not always included in animal models of ALI. Considering animal welfare and the 3Rs, mice receiving PBS with no cells were used as the only control group in our study. Second, MSCs were given at only one time point. In the present study, we aimed to evaluate the activation of MyD88–NFκB signaling which is an early immune response to infections and tissue damage. Therefore, we designed to administer MSCs to mice at one hour after LPS-induced ALI. There may be some discrepancy in the effects of MSCs if the time point changed. It remains an important issue to determine the optimal time point of MSC treatment for patients with ALI in a real-life scenario. Third, we did not give consideration to the issue that MSCs may have some different characteristics depending on the donors and other conditions. Although important for experimental studies and clinical utility, how to ameliorate the bio-diversity still remains a question.

## 4. Materials and Methods

### 4.1. Isolation of MSCs from Umbilical Cords

This study was approved by the Institutional Review Board of the Chung Shan Medical University Hospital (CS 14103), and written informed consents were obtained from the donors. UCMSCs were collected, isolated, and identified as in our previous reports [25,26,35,36]. Briefly, umbilical cords were obtained from full-term infants immediately after birth. The cord blood vessels were removed carefully to retain Wharton’s jelly, which was digested with collagenase and then placed in the culture medium (high-glucose DMEM with 10% fetal bovine serum). The cells were incubated at 37 °C in a humidified atmosphere under 5% CO_2_. The medium with suspension of non-adhered cells was discarded after 48 h and thereafter replaced twice a week. Upon reaching 80–90% confluence, the cells were detached with trypsin-EDTA (Gibco, Carlsbad, CA, USA) and re-plated for subculture. The cultured MSCs of passage 5 were used for further studies.

### 4.2. Identification of UCMSCs

The criteria of International Society for Cellular Therapy were used to characterize UCMSCs [37]. To evaluate surface marker expression, cultured UCMSCs were detached, washed, and resuspended in PBS. After fixing and blocking, the cells were immunolabeled with fluorescein isothiocyanate or phycoerythrin-conjugated mouse antihuman antibodies specific to CD34, CD45, CD14, CD73, CD90, or CD105. Nonspecific mouse IgG served as isotype control. All reagents were purchased from BD Biosciences. Data were analyzed by flow cytometry (FACSCalibur; BD Biosciences, San Jose, CA, USA) with CellQuest software.

To assess differentiation potential, cultured UCMSCs were detached and replated in 60 mm dishes. To promote adipogenic differentiation, UCMSCs were incubated in DMEM with 10% fetal bovine serum, 1 μM dexamethasone, 0.5 mM 3-isobutyl-1-methylxanthine (Sigma, St. Louis, MO, USA), 0.1 mM indomethacin (Sigma, St. Louis, MO, USA), and 10 μg/mL insulin (Novo Nordisk A/S, Bagsværd, Denmark). After 2 weeks, adipogenesis was confirmed by intracellular accumulation of lipid droplets stainable with oil red O (Sigma, St. Louis, MO, USA). To induce osteogenic differentiation, UCMSCs were grown in DMEM with 10% fetal bovine serum, 10 mM *β*-glycerophosphate (Sigma, St. Louis, MO, USA), 0.1 μM dexamethasone, and 0.2 mM ascorbic acid (Sigma, St. Louis, MO, USA). After 2 weeks, osteogenesis was demonstrated by mineralized deposits stainable with von Kossa stain (Cedarlane, ON, Canada).

### 4.3. LPS-Induced ALI in Mice

The experimental protocol was approved by the Institutional Animal Care and Use Committee of the Chung Shan Medical University Experimental Animal Center (IACUC Approval No: 1598). Eight-week-old female C57BL/6 mice were provided by the BioLASCO Taiwan and maintained in a temperature- and humidity-controlled environment with free access to food and water for two weeks before the experiment. LPS-induced ALI was performed after being anesthetized by the inhalation of isoflurane vapor mixed with oxygen. Mice were suspended by their cranial incisors with the tongue extracted to prevent swallowing reflex. LPS from *Escherichia coli* O55:B5 (Sigma-Aldrich, St. Louis, MO, USA) at the dose of 15 mg/kg was pipetted into the deep throat, and the nares were pinched to enhance liquid aspiration. Mice were randomly divided into two groups, and there were eight mice in each group. Mice of the MSC group (*n* = 8) received intraperitoneal injections of one million MSCs in 0.5 mL sterile PBS (Gibco, Gaithersburg, MD, USA) at one hour after LPS challenge. Mice of the control group (*n* = 8) received sterile PBS in a volume of 0.5 mL with no cells at the same time point.

### 4.4. Collection of Samples

To determine the initial changes in levels of circulating cytokines, blood samples were harvested from retro-orbital sinus bleeding at 6 h after LPS challenge. Then, all mice were sacrificed at 48 h after LPS-induced ALI. Bronchoalveolar lavage was performed after anesthetization with intramuscular injection of ketamine (75 mg/kg) and xylazine (5 mg/kg). A 20-gauge catheter was placed into the trachea through which 1 mL of PBS was flushed back and forth five times. The BALF was collected, and the volume was recorded. The protein concentration in the BALF was measured by the Bradford assay (Bio-Rad, Hercules, CA, USA) in accordance with the manufacturer’s instructions. Finally, cardiac puncture was performed to obtain blood samples. The anterior chest wall was surgically removed, and bilateral lungs were excised.

### 4.5. Determination of Cytokine Levels in the Serum and BALF

To determine circulating cytokine levels, serum was separated by centrifugation at 10,000× *g* for 10 min immediately after collection. The concentrations of IL-6, TNF-α, MCP-1, IL-1β, and IL-8 in the serum and BALF were measured separately by cytometric bead array immunoassay (BD CBA Mouse Soluble Protein Flex Set System; BD Biosciences, San Jose, CA, USA), according to the manufacturer’s instructions. Data were analyzed using flow cytometry (FACSCanto; BD Biosciences, San Jose, CA, USA) with the FCAP array software. Reactions were performed in duplicate for the serum and triplicate for the BALF.

### 4.6. Lung Histopathology and Immunohistochemistry

The harvested lungs were fixed in 10% formalin for 24 h. Then, the specimens were embedded in paraffin and stained with hematoxylin and eosin for histologic assessment. Two pathologists independently evaluated the pathologic changes and graded the degree of lung injury based on the semiquantitative scoring system. Seven main characteristics were used as scoring parameters, including proportion of intra-alveolar macrophages in the infiltrate, septal edema, congestion, degree of neutrophil infiltration, septal mononuclear cell infiltration, alveolar hemorrhage, and alveolar edema [38]. Each parameter was scored from 0 to 4 (0 = normal, 4 = most severe). The total score was obtained by adding the values for each parameter for each animal.

For immunohistochemical analysis, the lung tissue sections from deparaffinized specimens were stained with anti-MyD88 antibody (Gene Tex, GTX112987) or anti-phospho-NFκB p65 antibody (Gene Tex, GTX50254). The UltraVision Quanto Detection System (Thermo Fisher Scientific, Waltham, MA, USA) was used to amplify the signal. The immunostaining was visualized with 3,3-diaminobenzidine and counterstained with hematoxylin (Sigma, St. Louis, MO, USA).

### 4.7. Assessment of MyD88-NFκB Activation by Western Blot Analysis

After lysis, lung tissue samples (50 μg) were run on 12.5% sodium dodecyl sulfate polyacrylamide gels and transferred to polyvinylidene fluoride membranes. After blocking with 5% bovine serum albumin in Tris-buffered saline, the membranes were incubated overnight at 4 °C with primary antibodies, anti-MyD88 (Gene Tex, GTX112987), or anti-phospho-NFκB (Gene Tex, GTX50254). Then, the membranes reacted with horseradish peroxidase-conjugated secondary antibody (Gene Tex, GTX213110-01) at room temperature for 1 h. As a loading control, the same blots were re-probed with anti-GAPDH (Gene Tex, GTX100118) and anti-mouse horseradish peroxidase antibodies. All samples were performed in triplicate.

### 4.8. Statistical Analysis

Data analysis was performed using SPSS 16.0 for Windows. For continuous variables, the Mann–Whitney U test was used to compare groups. A value of *p* < 0.05 was considered to be statistically significant.

## 5. Conclusions

Results from this study demonstrated potent immunomodulatory effects from intraperitoneal administration of human UCMSCs in wild-type mice exposed to LPS, which is a well-characterized animal model of ALI. MSCs effectively alleviated the surge of inflammatory cytokines and attenuated lung injury. For the first time, we documented that MSCs exerted their immunomodulatory influence on mice with ALI through down-regulation of the MyD88–NFκB signaling pathway. As a promising source of MSCs for clinical use, our data suggested that the systemic administration of UCMSCs can be a viable treatment option for human ALI. Further clinical trials are warranted.

## Figures and Tables

**Table 1:** Average lung injury score for each estimated feature.

	Control Group	MSC Group
Proportion of intra-alveolar macrophages in the infiltrate	2	1
Septal edema	1	0
Congestion	0.375	0.375
Degree of neutrophil infiltration	2.875	1.125
Septal mononuclear cell infiltration	1	1
Alveolar hemorrhage	0	0
Alveolar edema	0	0
Total lung injury score	7.25	3.50

Individual scores range from 0 to 4 (0 = normal, 4 = most severe). *n* = 8 mice/group.

## Abbreviations

ALI, acute lung injury; BALF, bronchoalveolar lavage fluid; IL, interleukin; LPS, lipopolysaccharides; MCP-1, monocyte chemoattractant protein-1; MSCs, mesenchymal stem cells; MyD88, myeloid differentiation factor 88; NFκB, nuclear factor-κB; PBS, phosphate-buffered saline; TLRs, Toll-like receptors; TNF-α, tumor necrosis factor-α; UCMSCs, umbilical cord-derived mesenchymal stem cells.
